# Supplementary material for: Evaluation of the environmental polio surveillance system—Northern Region, Ghana, 2021
Source: PLoS One. 2024 Feb 29;19(2):e0294305. doi: 10.1371/journal.pone.0294305 (PMC10903872; doi:10.1371/journal.pone.0294305)
Supplement: S3 File — (DOCX) [file pone.0294305.s003.docx]

**ENVIRONMENTAL POLIO VIRUS SURVEILLANCE SYSTEM EVALUATION CHECKLIST**

***Identification Information***

Region………………………. District………………………….. Date of interview…………………...

Sub-district………………………………………..Site…………………………….

Name of Respondent………………………………………………………………..

Cadre of respondent……………………………..Contact number……………….

***Availability of surveillance documents.***

1. Do you have the national guideline for environmental polio virus surveillance? a. Yes b. No c. Don’t know
2. If No, why? ……………………………………………………
3. Is there available guideline for sample collection, handling and transportation to the next level? a. Yes b. No c. Don’t know
4. If No, why? ………………………………………………………………………………
5. Are there reporting forms in this unit? a. Yes b. No c. Don’t know
6. If No, why? .....................................................................................................................

**OPERATION OF THE SYSTEM**

***Purpose and objectives of the system***

1. What is the purpose of the EPVS Surveillance System? ………………………………….
2. What are the objectives of the EPVS surveillance system………………………………..
3. What is the data collected used for? ………………………
4. Who uses your data? ……………………………………………

***What is the population under surveillance by this system?***

1. Describe the catchment population for this surveillance system (drainage system coverage)? ***How are samples collected and transported?***
2. What sample is collected? ...............................................................................................
3. How frequently do you collect samples? ………………………………………………………………………
4. When in the day do you collect samples and why? ……………………………………….
5. How are samples collected? …………………………………
6. How do you package the collected samples? ............................................................................
7. How are samples transported? …………………………………………………

**How are the system’s data collected?**

1. What are the sources of data collection?.................................
2. What period/time is data collected?………………………
3. What data are collected and how are they collected (***use reporting form as a guide***)?
4. Who collect the data?.....................................

***How are the system’s data managed?***

1. Data transfer………………………………………………
2. Data entry……………………………………………
3. Data editing…………………………………
4. Data storage……………………………………………….........
5. Back up of data………………………………………………
6. Data analysis……………………………………………………
7. How do you ensure privacy & confidentiality of data? ..........................
8. Is data quality check or validation conducted? a. Yes b. No c. Don’t know
9. If yes, how is it done, and who does it? (***Show evidence***) …………………………..

**How are the *system’s* data disseminated?**

1. To whom is data disseminated?……………………………………….......
2. How is data disseminated?……………………… …………
3. How do you get feedback?....................................................
4. How often do you get feedback......

***How is the system integrated with other systems in terms of:***

1. Structures used………………………….........
2. Personnel used…………………………………………….
3. Funding……………………………………………………
4. Data reporting system……………………....................
5. Notification for action……………………..............................

***Draw a flow chart of the system***

1. Adapt an available system’s flow chart if any …..………………..……………………………………….OR
2. Develop flow chart with the assistance of the Regional Surveillance Officer ………

***Resources used to operate the surveillance system:***

1. What is the source of funding for the surveillance system? …………………………………………………
2. By, estimates, how much does the following cost the system in a month?
3. Travel …………………………………………………………………….
4. Training …………………………………………………………………..
5. Supplies …………………………………………………………………
6. Computer and other equipment ………………………………………….
7. Mail & Telephone ……………………………………………………….
8. Internet connections ……………………………………………………..
9. Laboratory support ………………………………………………………
10. Hardware and software maintenance ……………………………………
11. Any other cost (specify) …………………………………………………
12. Estimate the time it takes to operate the system, including the collection, editing, analysis, and dissemination of data ………………………………………………….

**Simplicity**

1. How is the surveillance system data collected? …………………………………………………………………………………
2. How is the surveillance system data recorded? ……………………………………………
3. How many forms per sample are filled? ……………………………………………
4. How is the surveillance system data reported to the next level? ………………………
5. How long (minutes) does it take to complete recording information on a sample collected? ……………………………………………
6. How often are data been reported to the next level? ………………………………………
7. Does the system have a flow chart? a. Yes b. No c. Don’t know
8. If yes, how simple is the flow chart? ……………………………………………

**Flexibility**

1. Has there been any change(s) to the system in the past? a. Yes b. No c. Don’t Know
2. If yes, what do you think influenced the change(s)? …………………………
3. Did the change(s) affected the system’s functionality? a. Yes b. No c. Don’t Know
4. If yes, how did it affect the system? …………………………………………
5. If no changes, do you think any change(s) in the existing procedure of sample collection and reporting formats will affect the . a. Yes b. No c. Don’t Know
6. If yes, how? ……………………………………………………………
7. Is the surveillance system easy to integrate with other systems? a. Yes b. No c. Don’t Know
8. If no, why? ………………………………………………
9. Is the system easy to add new information technology? a. Yes b. No c. Don’t Know
10. If no, why? …………………………………………………………………

**Data quality (Records review)**

1. Are all reported forms completely filled? a. Yes b. No
2. If No, what is the proportion of uncompleted forms for the last two years? ………………………………...
3. Is the recorded data clear to read and understand? a. Yes b. No
4. If No, what is the proportion of records that are not clear for the last two years? …………………………
5. Check for consistency in data at the various levels. ………………………………………………………………
6. Do you do data validation? a. Yes b. No
7. If yes, show evidence. …………………………………………
8. If no, why? ……………………………………………………....

**Stability**

1. Is the sewage/waste water flowing in the sewage system all year round? a. Yes b. No
2. Was there lack of resources that interrupt the surveillance system? a. Yes b. No
3. Was there any time /condition in which the surveillance is not fully operating? a. Yes b. No
4. If yes, explain why? …………………………………………………………....
5. What are the challenges faced by the system in the following areas

Expertise ………………………………………………

Transport …………………………………………………...

Electricity ………………………………….

Funding …………………………………….

**Acceptability**

1. How many of the expected reporting sites are sending reports? ………………
2. How many reporting sites sent in complete reports? …………………………
3. How many reporting sites sent in reports on time? …………………………………
4. Were all the reporting agents sending their report using the current and appropriate surveillance-reporting format? a. Yes b. No
5. If No, why? ……………………………………………………………………………………………
6. What difficulties are there in filling the surveillance forms? …………………

**Representativeness**

1. Is the sewage/waste water system collecting sewage/waste water from the whole catchment area?

a. Yes b. No c. Don’t Know

1. If No, why? ……………………………………………………........
2. Are both surveillance sites collecting samples on scheduled? a. Yes b. No c. Don’t Know
3. If No, why? ……………………………………………....

**Sensitivity**

1. Has the system detected outbreak before? a. Yes b) No
2. If yes, how many? ...................................
3. How were these outbreaks detected? ……………………………………..
4. If No outbreak was detected, why? …………………………………………………….

**Timeliness**

1. How long (minutes) does it take to record information on the sample collection form? …
2. How long does it take for the collecting sites to submit reports to the district/region? …
3. How long does it take for laboratory confirmation? …………………………
4. How long does it take for feedback from the next level? …………………………
5. Manually review data for the timeliness of reporting ………………………………

**Usefulness**

1. Has there been isolation of polio virus in any of the samples collected for the past two years? a. Yes b. No c. Don’t Know
2. If yes, what action was taken? …………………………………………………………

If no, what action will be taken if polio virus is isolated from future samples? ………
