# Supplementary material for: Evaluation of the environmental polio surveillance system—Northern Region, Ghana, 2021
Source: PLoS One. 2024 Feb 29;19(2):e0294305. doi: 10.1371/journal.pone.0294305 (PMC10903872; doi:10.1371/journal.pone.0294305)
Supplement: Attachment — Submitted filename: Response to Reviewers.docx [file pone.0294305.s006.docx]

**Journal Requirements:**

| **Comment** | **Response** |
| --- | --- |
| 1. Please ensure that your manuscript meets PLOS ONE's style requirements, including those for file naming. The PLOS ONE style templates can be found at  <https://journals.plos.org/plosone/s/file?id=wjVg/PLOSOne_formatting_sample_main_body.pdf> and  <https://journals.plos.org/plosone/s/file?id=ba62/PLOSOne_formatting_sample_title_authors_affiliations.pdf> |  |
| 2. We note that Figure 1 in your submission contain copyrighted images. All PLOS content is published under the Creative Commons Attribution License (CC BY 4.0), which means that the manuscript, images, and Supporting Information files will be freely available online, and any third party is permitted to access, download, copy, distribute, and use these materials in any way, even commercially, with proper attribution. For more information, see our copyright guidelines: <http://journals.plos.org/plosone/s/licenses-and-copyright>.  We require you to either (1) present written permission from the copyright holder to publish these figures specifically under the CC BY 4.0 license, or (2) remove the figures from your submission:   a. You may seek permission from the original copyright holder of Figure 1 to publish the content specifically under the CC BY 4.0 license.    We recommend that you contact the original copyright holder with the Content Permission Form (<http://journals.plos.org/plosone/s/file?id=7c09/content-permission-form.pdf>) and the following text: “I request permission for the open-access journal PLOS ONE to publish XXX under the Creative Commons Attribution License (CCAL) CC BY 4.0 (<http://creativecommons.org/licenses/by/4.0/>). Please be aware that this license allows unrestricted use and distribution, even commercially, by third parties. Please reply and provide explicit written permission to publish XXX under a CC BY license and complete the  attached form.”   Please upload the completed Content Permission Form or other proof of granted permissions as an "Other" file with your submission.    In the figure caption of the copyrighted figure, please include the following text: “Reprinted from [ref] under a CC BY license, with permission from [name of publisher], original copyright [original copyright year].”   b. If you are unable to obtain permission from the original copyright holder to publish these figures under the CC BY 4.0 license or if the copyright holder’s requirements are incompatible with the CC BY 4.0 license, please either i) remove the figure or ii) supply a replacement figure that complies with the CC BY 4.0 license. Please check copyright information on all replacement figures and update the figure caption with source information. If applicable, please specify in the figure caption text when a figure is similar but not identical to the original image and is therefore for illustrative purposes only. | We are grateful for the comment. Figure 1 has been removed from the revised manuscript |
| 3. In your Data Availability statement, you have not specified where the minimal data set underlying the results described in your manuscript can be found. PLOS defines a study's minimal data set as the underlying data used to reach the conclusions drawn in the manuscript and any additional data required to replicate the reported study findings in their entirety. All PLOS journals require that the minimal data set be made fully available. For more information about our data policy, please see <http://journals.plos.org/plosone/s/data-availability>.  "Upon re-submitting your revised manuscript, please upload your study’s minimal underlying data set as either Supporting Information files or to a stable, public repository and include the relevant URLs, DOIs, or accession numbers within your revised cover letter. For a list of acceptable repositories, please see  <http://journals.plos.org/plosone/s/data-availability#loc-recommended-repositories>. Any potentially identifying patient information must be fully anonymized.  Important: If there are ethical or legal restrictions to sharing your data publicly, please explain these restrictions in detail. Please see our guidelines for more information on what we consider unacceptable restrictions to publicly sharing data: <http://journals.plos.org/plosone/s/data-availability#loc-unacceptable-data-access-restrictions>. Note that it is not acceptable for the authors to be the sole named individuals responsible for ensuring data access.  We will update your Data Availability statement to reflect the information you provide in your cover letter. |  |
| 4. Please include a separate caption for each figure in your manuscript. | We are grateful for the comment. We have include separate captions for each Figure and the Table. Details:  Figure 1: page 6, line 142  Figure 2: page 8, line 192  Figure 3: page 11, line 256  Figure 4: page 11, line 258  Table 1: page 11, lin2 262 |
| 5. Please include captions for your Supporting Information files at the end of your manuscript, and update any in-text citations to match accordingly. Please see our Supporting Information guidelines for more information: <http://journals.plos.org/plosone/s/supporting-information>.  [Note: HTML markup is below. Please do not edit.] |  |

**Reviewer 1:**

| **Comment** | **Response** |
| --- | --- |
| The manuscript is well written though a few typo and grammatical errors have been indicated in the attached document. | We are grateful for the corrections made to the typo and grammatical errors. We accept all the corrections made in the revised manuscript |
| **Other technical issues have also been raised in the document that will require the authors to address them:** | We are grateful for these technical issues raised. The manuscript has been revised to address all the issues. The location of the details are indicated against each comment: |
| Line 190, What tool was used in analyzing the qualitative data? Indicate in the data analysis and presentation section.  Line 190, Indicate the thematic areas involved in the analysis | Page 10, lines 227-237 |
| Line 192, Please indicate the company name and the location of the company that designed the software  Eg. Microsoft Excel version 16 (Microsoft Inc., USA) | Page 9, lines 213-214 |
| Line 219, “All the respondents knew……………” Indicate the numbers and proportions | Page 12, line 268 |
| Line 294: The reference cited does not correlate with this statement. The citation is for a study conducted in Pakistan, not Egypt. Please cite appropriately | Page 15, line 344 |

**Reviewer 2:**

| **Comment** | **Response** |
| --- | --- |
|  | We are grateful for these comments. The manuscript has been revised to address all the comments. The location of the details are indicated against each comment: |
| 55-56. Maybe add reasons for the global decrease in polio reports since 1988. | Page 3, lines 55-56 |
| 62. Neighbouring countries from the southern or northern part. I want to assume these outbreaks came from northern part of Ghana? 94. Northern region | Pages 3&4, Lines 68-74 |
| 335. What parameter are you using to determine delays. Is there a stipulated time that could be used to measure this? It would have been good if data is shown on national level delays a bit.  335-336. Is the quarterly submission of results from national or Nogushi the norm? Did you find out? | We deleted this and captured it as a limitation  Details: page 17, line 387-389 and lines 403-404 |
| 348. All year round | Page 17, lines 400-401 |

**Reviewer 3:**

| **Comment** | **Response** |
| --- | --- |
| **Major comments** |  |
| It would be very helpful to the reader to include a map of the Northern region showing the location of the two ES sites and the corresponding drainage settlements, and the location of the cVDPV2 AFP case. | We are grateful for the comment. We have included the map (Figure 1, page 6) as suggested. |
| It is not clear if the ES sites pass the WHO’s ES performance criteria (Table 5 of <http://polioeradication.org/wp-content/uploads/2022/11/Field-Guidance-for-the-Implementation-of-ES-20221118-ENG.pdf>) This is essential. It is important to document the detection of enterovirus and Sabin strains from ES sites but this is currently absent from the paper. The sites should be assessed whether they meet the WHO criteria. | We are thankful for the comment. We have revised the manuscript to reflect this comment.  Details: pages 9&10, lines 215-226 and Table 1, page 11 |
| **Minor comments** |  |
| **Abstract** | We are grateful for these comments. The manuscript has been revised to address all the comments in this section. The location of the details are indicated against each comment: |
| Abstract: please include the precise dates of the study period | Details: page 2, line 34 |
| Abstract: The version of Excel seems excessive for the abstract | Details: page 2, line 35 |
| Abstract: ‘Feedbacks were not regular’ – can you quantify this statement. What proportion of results took longer than a month or another appropriate timeframe? Feedback to who? | We deleted this and captured it as a limitation  Details: page 2, line 42 and page 17, lines 403-404 |
| **Introduction** | We are grateful for these comments. The manuscript has been revised to address all the comments in this section. The location of the details are indicated against each comment: |
| Line56: 1988 – it would be clearer if you explained this is when the Eradication Initiative began. | Page 3, lines 55-56 |
| Line 57 No wild type 3 Poliovirus since October 1999 in incorrect. Please correct this | Page 3, lines 58-61 |
| Line 60: The last case of wild polio in Nigeria was in 2016 not 2014 | Page 3, lines 67-58 |
| Line 64: Please define what vaccine derived poliovirus is | Page 3, lines 61-64 |
| Lines 81-82: Unfortunately seems the wrong choice of word. Also poliovirus isn’t in circulation in the environment (it’s detection can indicate circulation but it does not replicate in the environment). | Page 4&5, lines 93-97 |
| Line 94: Northern region. Where else in the country is ES implemented? Why is the Northern region important? Because of historic WPV circulation? | Page 5, lines 106-107-56 |
| **Methods** | We are grateful for these comments. The manuscript has been revised to address all the comments in this section. The location of the details are indicated against each comment: |
| Line 116 – how were the site locations selected?  Line 117 – what methods were used to assess the location of the drainage settlements? Was the sewage network mapped? | Page 6, lines 128-142 |
| Line 168: ‘Stool turns positive’ I think you mean tests positive. A stool doesn’t turn positive | Page 8, line 189 |
| Line 156: What do you mean by ‘results are sent to the region’. Who in the region? Are they also sent to national representatives at the same time? | We deleted this and captured it as a limitation  Details: page 8, line 177 and page 17, lines 403-404 |
| Line 131 – It would be helpful to include the questionnaire in the appendix |  |
|  |  |
| **Results** | We are grateful for these comments. The manuscript has been revised to address all the comments in this section. The location of the details are indicated against each comment: |
| Line 227 – what are the other priority diseases? | Page 12, lines 176-178 |
| Line 255 – delayed by how long? | We deleted this and captured it as a limitation  Details: page 8, line 177 and page 17, lines 403-404 |
| Figure 3: Positive for what – cVDPV2? It would be interesting to show the number positive for Sabin 1,2,3 and non-polio enterovirus as well | Figure 3 |
| Figure 4 – Suspected – do you mean non-polio AFP? Confirmed, confirmed for what? cVDPV2? It would be helpful to show the timings of the vaccination campaigns. | Figure 4 |
| **Discussion** |  |
| Line 277 – ES is faster – do you mean in this setting or more generally? If the latter please provide a reference. | Page 14, lines 328-329 |
| Line 279 – On average, this is an average over what? | Page 14, line 331 |
