# Supplementary material for: Evaluation of the environmental polio surveillance system—Northern Region, Ghana, 2021
Source: PLoS One. 2024 Feb 29;19(2):e0294305. doi: 10.1371/journal.pone.0294305 (PMC10903872; doi:10.1371/journal.pone.0294305)
Supplement: Attachment — Submitted filename: Response to Reviewers.docx [file pone.0294305.s007.docx]

**Reviewer comment:**

| **COMMENT** | **RESPONSE** |
| --- | --- |
| 1) The authors state that they follow the CDC guidelines for evaluating a surveillance system but they have excluded some critical assessments such as sensitivity and predictive value positive. | We are grateful for the comment. We have revised the manuscript to provide information on sensitivity and predictive value positive  Details: Page 2, lines 44-45  Page 8, lines 174-178  Page 14, lines 332-336  Page 18, lines 422-423 |
| 2) The population of the area under surveillance is 2.3million yet the sampling is done for a population of about 70,000 people, short of the 100,000 to 300,000 recommended for global polio surveillance. It is unclear how this sampling location was selected. Is this sample size sufficient for ongoing surveillance and monitoring? | We are grateful for the comment. We initially only considered communities around the sample collection sites. However, other communities at the upper part of the sample collection sites feed into the drain and sewer lines but were not classified as drainage settlement. These communities have been added making the total population to be 102,653 (70,310 for Nyanshegu and 32,343 for Koblimahgu).  Details: Page 6, lines 128-133  In addition to the population (102,653), the sampling location was selected based on four criteria  Details: Page 6, lines 122-126 |
| 3) For the subjective aspects of the evaluation, little data was shared regarding staff surveys/interviews regarding their feedback e.g. no summary of responses to specific questions though a data collection tool was mentioned. | We are grateful for the comment. We have revised the manuscript to include some direct quotations from respondents  Details: Page 12 - 15, lines 270-349 |
| 4) Other factors to consider in evaluating wastewater surveillance particularly where there is a low risk of wild poliovirus or vdpv include the detection of Sabin strains from OPV use (i.e. correlation with immunization days) and non-polio enteroviruses (NPEV) used as proxy indicators to regularly monitor, validate, and compare site sensitivity. These indicators were not assessed. | We are grateful for the comment.  The manuscript has been revised to include results on NPENT, Sabin and NPENT+Sabin viruses detected and were used in calculating the sensivity.  Details:  Page 14, lines 332-336  Page 10 - 11, lines 244 - 246  Figure 3 |
